# Body Composition Analysis Allows the Prediction of Urinary Creatinine Excretion and of Renal Function in Chronic Kidney Disease Patients

**DOI:** 10.3390/nu9060553

**Published:** 2017-05-28

**Authors:** Carlo Donadio

**Affiliations:** Department of Clinical and Experimental Medicine, Division of Nephrology, University of Pisa, I-56100 Pisa, Italy; carlo.donadio@med.unipi.it; Tel.: +39-050-99-7278

**Keywords:** glomerular filtration rate, 24-h urinary creatinine excretion, creatinine clearance, estimate of renal function, body composition analysis, body cell mass, electrical body impedance

## Abstract

The aim of this study was to predict urinary creatinine excretion (UCr), creatinine clearance (CCr) and the glomerular filtration rate (GFR) from body composition analysis. Body cell mass (BCM) is the compartment which contains muscle mass, which is where creatinine is generated. BCM was measured with body impedance analysis in 165 chronic kidney disease (CKD) adult patients (72 women) with serum creatinine (SCr) 0.6–14.4 mg/dL. The GFR was measured (^99m^Tc-DTPA) and was predicted using the Modification of Diet in Renal Disease (MDRD) formula. The other examined parameters were SCr, 24-h UCr and measured 24-h CCr (mCCr). A strict linear correlation was found between 24-h UCr and BCM (*r* = 0.772). Multiple linear regression (MR) indicated that UCr was positively correlated with BCM, body weight and male gender, and negatively correlated with age and SCr. UCr predicted using the MR equation (MR-UCr) was quite similar to 24-h UCr. CCr predicted from MR-UCr and SCr (MR-BCM-CCr) was very similar to mCCr with a high correlation (*r* = 0.950), concordance and a low prediction error (8.9 mL/min/1.73 m^2^). From the relationship between the GFR and the BCM/SCr ratio, we predicted the GFR (BCM GFR). The BCM GFR was very similar to the GFR with a high correlation (*r* = 0.906), concordance and a low prediction error (12.4 mL/min/1.73 m^2^). In CKD patients, UCr, CCr and the GFR can be predicted from body composition analysis.

## 1. Introduction

An accurate evaluation of renal function is important in chronic kidney disease (CKD) patients to classify the stage of CKD, to evaluate the progression of kidney impairment and to prevent toxic effects due to inappropriate dosage of drugs. The “gold standard” method to assess renal function is the direct measurement of the glomerular filtration rate (GFR) from the clearance of inulin, or of other glomerular tracers [1]. However, in clinical practice, renal function is commonly evaluated by serum creatinine (SCr) or creatinine clearance (CCr). SCr is a very simple and highly reproducible test. Unfortunately, its sensitivity is quite poor. Furthermore, SCr levels are influenced by the amount of muscle mass. On the other hand, the usefulness of CCr is greatly reduced by the high variability of its measurement, mainly due to the difficulty in obtaining an accurate collection of 24-h urine [2,3]. To simplify the procedure and to avoid urine collection, different formulas have been proposed to predict CCr or the GFR from serum creatinine (SCr) and anthropometric data [4,5]. The inaccuracy in the estimate of urinary creatinine excretion (UCr) is probably the major cause of error of the prediction formulas.

Total body electrical impedance analysis (BIA) is commonly used to evaluate body composition and the equilibrium of body fluid compartments in CKD patients [6,7,8,9]. The 24-h UCr is correlated to the amount of muscle mass [10,11]. Our previous data indicated that the value of body cell mass (BCM), which is the body compartment which encompasses muscle mass, is strictly correlated with 24-h UCr, and may allow the prediction of renal function [12,13,14].

The aim of this study was to evaluate the possibility of obtaining an accurate prediction of UCr and hence of CCr, and even the GFR from the measure of BCM obtained with BIA. First, we evaluated the relationship of UCr with BCM; then, we assessed the relevance of the other anthropometric determinants of UCr. From these data, we predicted UCr and hence CCr from SCr combined with BCM and other anthropometric data. Finally, we assessed the relationship between the measured GFR and the ratio of BCM/SCr. From this relationship, we developed a new formula to predict the GFR from BCM and SCr (BCM GFR).

## 2. Materials and Methods

### 2.1. Patients

Inclusion criteria: measurement of the GFR for the assessment of renal function in clinically stable adult CKD patients. Hypertensive patients and those at CKD stage 4–5 were on a salt restricted diet (≤2 g/day). No patients were treated with maintenance hemodialysis. Exclusion criteria: recent administration (within 2 weeks) of potentially nephrotoxic drugs or contrast media; acute kidney injury (AKI). CKD and AKI were defined according to National Kidney Foundation’s Kidney Disease Outcomes Quality Initiative guidelines.

**Figure 1:**
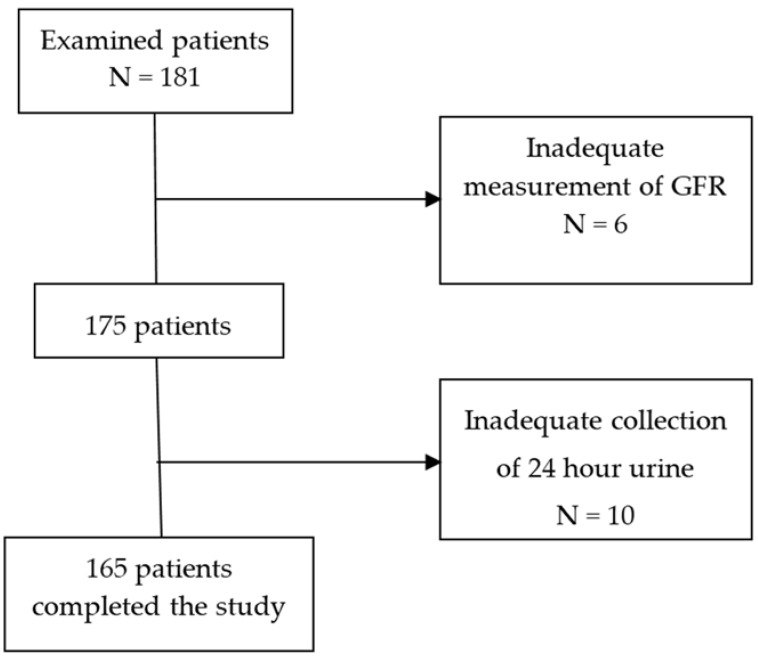
Flow diagram of the examined patients. GFR= glomerular filtration rate.

One hundred and eighty-one patients were examined and 165 patients entered the study. All patients were Caucasian (Figure 1). Diagnoses of kidney diseases were ischemic nephropathy (35%), interstitial nephritis (17%), glomerulonephritis (15%), unknown CKD (13%), diabetic nephropathy (10%), polycystic kidney disease (6%) and cancer (4%). The main clinical and anthropometric data of the patients analyzed in the present study are reported in Table 1. The study was approved by the Institutional Ethical Committee of Azienda Ospedaliero-Universitaria Pisana and conducted in accordance with the guidelines of the Helsinki declarations. All patients gave verbally their informed consent.

### 2.2. Methods

#### 2.2.1. Body Composition Analysis: Measurement of Body Cell Mass

Total body electrical impedance was measured with a tetrapolar impedance plethysmograph (BIA 109, Akern, Firenze, Italy) in patients lying supine, while fasting. Two electrodes were placed on the dorsal surface of the right hand and two on the dorsal surface of the right foot [7]. The analysis of single frequency electrical impedance (0.8 mA, 50 kHz) gives the values of resistance and reactance. BCM was calculated according to the manufacturer's equation from the values of resistance and reactance combined with body height and weight.

#### 2.2.2. Measurement and Prediction of Renal Function: 24-h Urinary Creatinine Excretion, Creatinine Clearance, Glomerular Filtration Rate

Patients were instructed to collect a 24-h urine sample on the day preceding GFR measurement. Urine volume was measured in our laboratory. A urine sample from the urine collection and a blood sample were drawn and immediately analyzed. Serum and urinary concentrations of creatinine were measured with a rate-blanked creatinine/Jaffé method traceable to an IDMS reference method (CREA Roche/Hitachi automated analysis for Hitachi 917, Roche Diagnostics, Mannheim, Germany; reference intervals for serum concentration are 0.50–0.90 mg/dL in women and 0.70–1.20 mg/dL in men). The 24-h urinary creatinine excretion (mg/24 h) was calculated as UCr (mg/dL) × urinary output in 24 h (dL). The 24-h creatinine clearance (mCCr) was calculated with the standard formula UCr (mg/dL) × UVol (mL/min)/SCr (mg/dL). CCr was predicted from BCM (see below) and, for comparison, by means of Cockcroft–Gault formula (C&G-CCr) [4].

The glomerular filtration rate (GFR) was measured in the morning before breakfast with a radio-isotopic method, as the urinary clearance of ^99m^Tc-DTPA [15,16]. This is a reference method for the measurement of the GFR. The GFR was also predicted using the Modification of Diet in Renal Disease four variables formula (MDRD GFR) [5] and with a newly developed formula (see below) from BCM and SCr (BCM GFR). Patients were classified in the different stages of CKD: CKD stage 1 *n* = 12; CKD stage 2 *n* = 55; CKD stage 3a *n* = 19; CKD stage 3b *n* = 31; CKD stage 4 *n* = 21; CKD stage 5 *n* = 27. Measured and predicted values of renal function are expressed as mL/min/1.73 m^2^.

### 2.3. Statistical Analysis

The normality of distribution of data was checked using the D’Agostino–Pearson test. Data are reported as mean ± standard deviation, or as median and interquartile range 25–75 (IQR 25–75) as appropriate. The significance of the differences between two independent samples was tested using the non-parametric Mann–Whitney tests or by means of a Student *t* test, as appropriate. The concordance correlation coefficient between predicted and measured values was tested [17]. The agreements between predicted and measured values were tested with Band and Altman plots [18]. The significance of the differences among correlation coefficients was tested [19]. Stepwise multiple regression analysis was used to establish the determinants of UCr excretion [20]. Mean prediction errors of predicted versus measured values were calculated [21]. Diagnostic accuracy was tested by Receiver Operating Characteristics (ROC) plot analysis [22]. Statistical analysis was performed mainly using MedCalc Statistical Software version 16.4.3 (MedCalc Software bvba, Ostend, Belgium; 2016) A *p* value < 0.05 was considered statistically significant.

## 3. Results

### 3.1. Anthropometric, Clinical and Laboratory Data: Body Composition, and Urinary Creatinine Excretion

**Figure 2:**
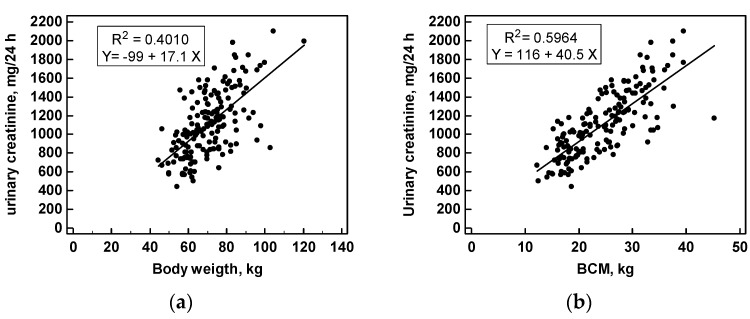
Correlations of 24-h urinary creatinine excretion (mg/24 h) with (**a**) body weight and (**b**) body cell mass (BCM). Parameters of linear regression and correlation coefficients *R*^2^ are reported.

Measures of body weight (BW), body surface area (BSA), body mass index (BMI), serum creatinine, 24-h urinary creatinine excretion, fat mass, extracellular water, BCM, and the ratios 24-h UCr/BW (mg/kg), and 24-h UCr/BCM (mg/kg) are reported (Table 1). Statistically significant differences were found between women and men in BW, BSA, fat mass, extra-cellular water, BCM, SCr, 24-h UCr, and for the ratio 24-h UCr/BW, while no differences were found in BMI and in the ratio of 24-h UCr/BCM. In fact, the mean ratio of 24-h UCr/BCM was 45.6 ± 9.0 mg/kg: 45.3 ± 9.5 in women and 45.8 ± 8.6 in men (*p* = 0.707). This ratio represents the milligrams of creatinine excreted in the 24-h urine per kilogram of BCM. The ratio UCr/BCM was independent from extra-cellular water volume. A closer linear correlation was found between 24-h UCr and BCM (*r*^2^ = 0.5964) than with body weight (*r*^2^ = 0.4010); the difference was statistically significant (*p* < 0.005) (Figure 2).

Multiple linear regression (MR) modeling for urinary creatinine excretion indicated that UCr was correlated positively with BCM, BW, and male gender, and negatively with age and SCr (Table 2). Height, body surface area and body mass index were not included in the model. The multiple correlation coefficient r was 0.8364, which was slightly, but not significantly higher (*p* = 0.0969) than the correlation coefficient (*r* = 0.7723) between 24-h UCr and BCM alone. A variance inflation factor of 4.582 indicated a moderate collinearity for BCM.

### 3.2. Predicted versus Measured Urinary Creatinine Excretion

**Figure 3:**
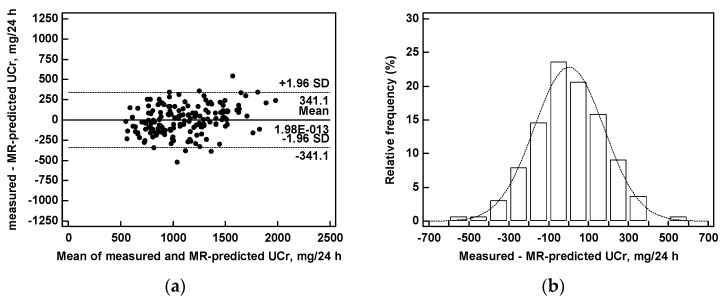
Measured versus predicted urinary creatinine excretion: (**a**) Agreement plot between measured 24-h urinary creatinine (UCr) and urinary creatinine predicted from BCM and multiple regression analysis equation (MR-predicted UCr), and (**b**) histograms of frequency distribution of the differences between measured and predicted UCr.

UCr was predicted from the multiple regression equation (MR-predicted UCr) and compared with 24-h measured UCr (Figure 3, Table 3). The correlation coefficient between the two measures was quite high (*r* = 0.8634). The linear regression equation between MR-predicted UCr and 24-h UCr was *Y* = 282 + 0.745*X* and the intercept and the slope values were significant (*p* < 0.0001). The agreement between predicted and measured UCr was satisfactory, with a mean difference of 2 mg; range of agreement from +341 to −341 mg. The agreement was similar in the whole range of measured UCr (from 444 to 2102 mg/24 h). The differences between predicted and measured UCr were normally distributed (Figure 3, Table 3). The mean prediction error was 175 mg. The concordance correlation coefficient between predicted UCr and measured UCr was 0.8545 (95% confidence interval 0.8089 to 0.8893). The difference between predicted and measured UCr was ≤30% in 92% and ≤15% in 62% of cases.

### 3.3. Predicted *versus* Measured Creatinine Clearance

Creatinine clearance was calculated from the predicted value of UCr (MR-predicted UCr) and from SCr as follows:

MR-BCM CCr (mL/min) = MR-predicted UCr (mg)/(SCr (mg/mL) × 1440 min)
(1)

For comparison, CCr was predicted also by means of C&G formula. The results of clearances were then reported to the standard BSA of 1.73 m^2^.

**Figure 4:**
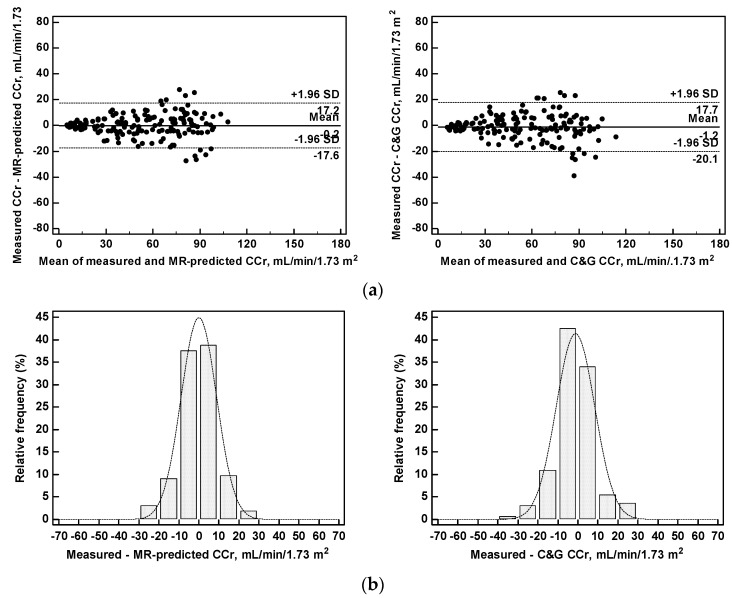
Measured versus predicted creatinine clearance: (**a**) Agreement plots between measured 24-h creatinine clearance (CCr) and creatinine clearance predicted from BCM and the multiple regression analysis equation (MR-predicted CCr). Creatinine clearance was predicted using the Cockcroft–Gault formula (C&G CCr); and (**b**) histograms of frequency distribution of the differences between measured and predicted clearances.

The concordance correlation coefficient of MR-BCM CCr with mCCr was quite high 0.9501 (c.i, 0.9327 to 0.9630), similar to that of C&G CCr 0.9424 (c.i. 0.926 to 0.9573).The agreement between predicted and measured clearances was also satisfactory: mean difference −0.2 mL/min/1.73 m^2^; range of agreement from +17.2 to −17.6 mL/min/1.73 m^2^ (MR-BCM CCr vs mCCr); mean difference −1.2 mL/min/1.73 m^2^; range of agreement from +17.7 to −20.1 mL/min/1.73 m^2^ (C&G CCr vs mCCr); the agreement between measured and predicted clearances remained similar in the whole range of measured CCr (from 5.4 to 109.6 mL/min/1.73 m^2^). The distribution of the differences between predicted and measured CCr was normally distributed around the 0 difference (Figure 4). The difference with measured CCr was ≤30% in 93% of patients with MR-BCM CCr and in 89% of patients with C&G CCr, and was lower than 15% in 64 and 60% of patients for MR-BCM CCr and C&G CCr, respectively. The mean prediction errors versus mCCr were also quite low and similar for MR-BCM CCr and C&G CCr (8.9 and 9.7 mL/min/1.73 m^2^, respectively, Table 3).

### 3.4. Predicted *versus* Measured Glomerular Filtration Rate

**Figure 5:**
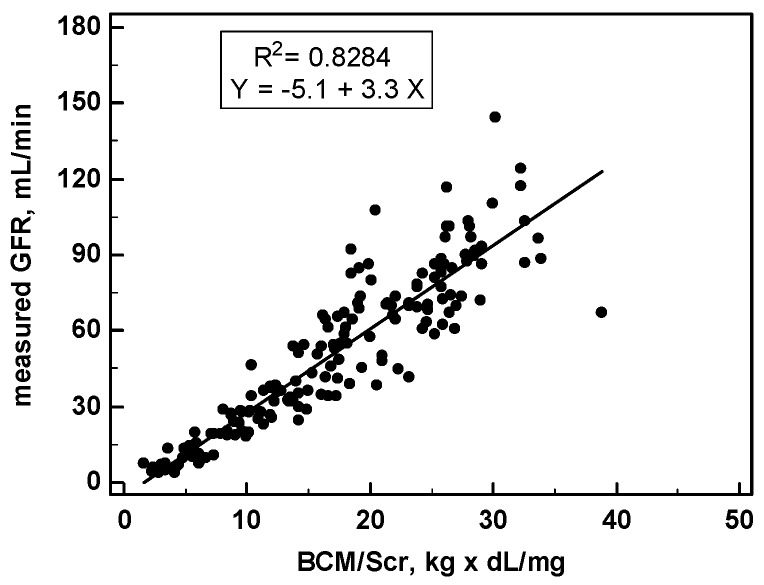
Correlation between glomerular filtration rate (GFR) and the ratio of body cell mass (BCM, kg) over serum creatinine (SCr, mg/dL).

A close linear correlation (*r* = 0.9102) was found between the GFR (measured as ^99m^Tc-DTPA clearance) and the BCM/SCr ratio in the 165 patients of this study (Figure 5).

This finding indicates that the value of the GFR can be estimated from individual values of BCM and SCr on the basis of the linear equation that links those parameters with the following formula: BCM GFR (mL/min) = −5.1 + 3.3 × BCM (kg)/ SCr (mg/dL). The results are then reported to the standard BSA of 1.73 m^2^. For comparison, the GFR was also predicted according to the IDMS-traceable MDRD 4 variables formula [5].

The concordance correlation coefficient of the BCM GFR with the GFR was quite high: 0.9007 (c.i, 0.8686 to 0.9252), similar to that of the MDRD GFR: 0.8989 (c.i. 0.8666 to 0.9239).

**Figure 6:**
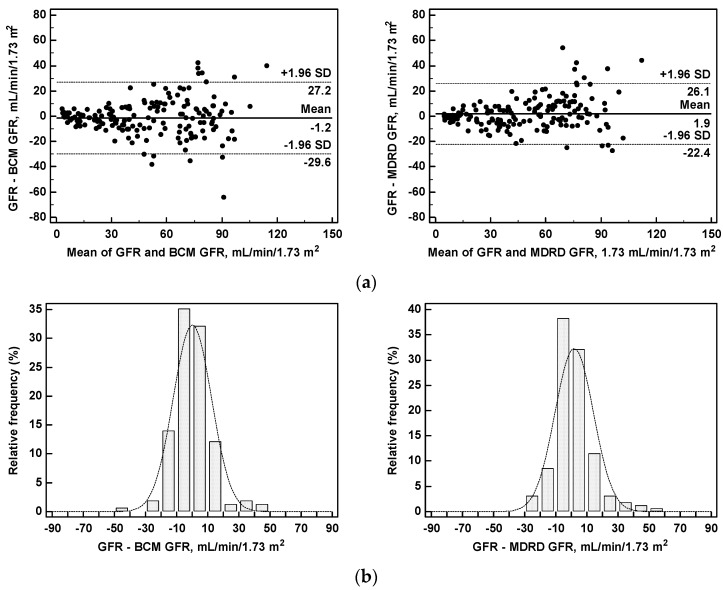
Measured versus predicted glomerular filtration rate (GFR): (**a**) Agreement plots between the measured GFR and the GFR predicted from BCM (BCM GFR) or from the MDRD formula (MDRD GFR), and (**b**) histograms of frequency distribution of the differences between the measured GFR and predicted values of GFR.

The agreement between the BCM GFR and the GFR was also satisfactory and similar to the MDRD GFR with a mean difference of −1.2 mL/min/1.73 m^2^, range of agreement from +27.2 to −29.6 mL/min/1.73 m^2^ (BCM GFR vs GFR); mean difference 1.9 mL/min/1.73 m^2^, range of agreement from +26.1 to −22.4 mL/min/1.73 m^2^ (MDRD GFR vs GFR). The differences between the BCM GFR and the GFR were normally distributed around the 0 difference (Figure 6).

The differences with measured GFR were ≤30% in 94 and in 92% of patients, and were ≤15% in 79 and 78% of patients for the BCM GFR and the MDRD GFR, respectively. The mean prediction errors versus the measured GFR were similar for the BCM GFR and the MDRD GFR (12.4 vs. 12.5 mL/min/1.73 m^2^, respectively). No differences in prediction errors were found between women and men (Table 3).

The accuracy of the BCM GFR as an indicator of different degrees of GFR (tested by ROC Plot analysis) was very high at all stages of CKD. AUC, sensitivity and specificity increased with the stage of CKD. No significant differences were found with the results of the MDRD GFR (Table 4).

## 4. Discussion

The early recognition of renal functional impairment may be useful to stop the development and progression of injury. For this purpose, there is a need for precise, accurate, reproducible and simple methods that are suitable for repeated measurements in order to assess renal function. Unfortunately, none of the methods currently used to evaluate the glomerular filtration rate fulfill these requirements. Inulin clearance is the gold standard to measure the GFR but it is not feasible in clinical practice. Other methods which measure the clearance of radioactive tracers, such as ^99m^Tc-DTPA [1,15], are precise and accurate but are expensive, somewhat complicated and not available everywhere. The measurement of serum creatinine concentration is the simplest method to evaluate renal function and has good reproducibility. However, its sensitivity as a marker of early impairment of renal function is quite low. Furthermore, due to its hyperbolic relationship with the GFR, the measurement of SCr allows only a gross estimation of the GFR. Finally, besides the level of the GFR, SCr also depends on the rate of creatinine production and on its volume of distribution. Consequently, it is influenced by the amount of muscle mass and total body fluids of the individual patients [23].

Due to the problems reported above, creatinine clearance remains the most commonly used test to measure renal function in clinical practice. However, the usefulness of CCr is greatly reduced by its low precision and accuracy. In order to reduce the variability of CCr measurements, due to incorrect collection of 24-h urine and to the variability of urinary creatinine excretion [3], different methods have been proposed to predict CCr and the GFR from SCr and some anthropometric data, thus avoiding urine collection. The method proposed by Cockcroft and Gault and the simplified four variables MDRD formula are probably the most frequently employed to predict 24-h CCr and the GFR [4,5]. However, in some groups of patients, such as obese, malnourished, edematous, elderly patients, or those with renal failure or liver disease, the predicted clearances do not completely agree with measured clearances or with the GFR, and the comparison with reference methods for the measurement of the GFR is often disappointing [24,25,26,27,28]. In particular, prediction errors of formulas are much higher in patients with better renal function. The inaccuracy in the prediction of urinary creatinine excretion (UCr) is probably the major cause of error in the prediction formulas. However, in some situations, prediction formulas are the only possible alternative to direct GFR measurement [29]. The measurement of total body electrical impedance is a simple and validated method to evaluate body composition and BCM in renal patients and in those with end-stage renal disease [6,8]. This measurement takes only a few minutes and is relatively convenient for the patient. The coefficient of variation (CV) of BCM measurements is very low (similar to the CV of BW measurements) [12]. 

The aim of this study was to evaluate the possibility of obtaining an accurate prediction of UCr, and hence of CCr, and even the GFR from the measure of BCM obtained with BIA. The limitations of this study are its monocentric nature and the need to validate the accuracy of the proposed prediction formulas in an external population of CKD patients. Furthermore, since some degree of extra-renal elimination of creatinine occurs in patients with advanced renal failure [30], it is expected that in CKD stage 5 patients, all the predictions of UCr, CCr and the GFR will be overestimates of the measured values. Furthermore, it was not possible to evaluate a possible effect of hydration status on serum creatinine levels and on the measure of BCM. The strengths of the study are the wide range of renal functions explored: from normality to advanced renal failure, and the measurement of the GFR with a gold standard method as a reference.

The results of this study performed in CKD patients at the different stages of GFR impairment demonstrate that an accurate prediction of creatinine excretion is possible from anthropometric data when the measurement of BCM is included in the prediction formula. In fact, UCr was more closely correlated to BCM than to BW. This result is in agreement with the fact that muscle mass, which is the compartment where creatinine is produced, represents the major constituent of BCM. We already demonstrated that the value of BCM is strictly correlated with creatinine excretion in CKD patients and with creatinine generation in maintenance haemodialysis (MHD) patients [9]. We found also that a low value of BCM is an indicator of poor nutritional status in CKD and MHD patients [31]. The results of the present study confirm also that CCr can be predicted accurately from the estimate of UCr obtained from BCM and anthropometric data [12,13]. The prediction error of MR-BCM CCr was quite low. The prediction formulas for urinary creatinine and creatinine clearance from BCM have also been validated by other authors [32]. Finally, our results indicate that it is also possible to accurately predict the GFR from the values of BCM and SCr, with a low prediction error, and confirm that the BCM GFR is a precise marker of the measured GFR (^99m^Tc-DTPA) [14]. Prediction errors of the BCM GFR and the MDRD GFR versus the measured GFR were quite similar, although the limits of agreement were sligthly wider for the BCM GFR. Our previous data indicate also that the interassay coefficient of variation of the GFR predicted from BCM is quite low (~9%) [14]. Due to its good agreement with the GFR, the high reproducibility of its measurement, its simplicity and low cost, the prediction of UCr, CCr and the GFR obtained from the measure of BCM is a feasible method for making repeated measurements of renal function. Our previous data in overweight and moderately obese CKD patients indicate that the BCM GFR has a closer agreement with the measured GFR than the MDRD GFR [33]. Furthermore, the values of BCM, extra-cellular water and fat-mass, obtained with BIA, allow the evaluation of nutritional status and of the equilibrium of body fluids, which is frequently affected in CKD patients [9,31].

## 5. Conclusions

In chronic kidney disease patients at the different stages of GFR impairment, urinary creatinine excretion may be accurately estimated and creatinine clearance and glomerular filtration rate may be accurately predicted to a satisfactory level of certainty from the value of body cell mass combined with other anthropometric data and with serum creatinine.

## Figures and Tables

**Table 1:** Main anthropometric, clinical, and laboratory data of the 165 patients. Median values and interquartile ranges (IQR 25–75) are reported. The statistical significance of the differences between women and men is indicated.

Parameters	All Patients *n* = 165			Women *n* = 72		Men *n* = 93		Significance
	Range	Median	IQR 25–75	Median	IQR 25–75	Median	IQR 25–75	*p*
Age, years	17–81	57.0	46–65	58.5	53–65	53.0	40.8–65.3	0.0142
Height, cm	143–188	1620	154–172	154	151–159	170	165–176	<0.0001
Body weight, kg	44.4–120.3	69.8	61.3–78.1	62.0	57.3–71.4	74.9	68.0–83.1	<0.0001
Body surface, m^2^	1.34–2.38	1.77	1.59–1.88	1.59	1.55–1.71	1.84	1.78–1.96	<0.0001
Body mass index, kg/m^2^	18.4–38.0	26.3	24.0–29.2	26.1	24.0–29.8	26.3	23.9–29.2	0.620
Serum creatinine, mg/dL	0.57–14.4	1.29	1.05–2.11	1.19	0.95–1.67	1.48	1.10–3.18	<0.0006
24-h UCr, mg	444–2102	1077	842–1337	883	738–1017	1270	1083–1506	<0.0001
Body cell mass, kg	12.2–45.2	24.4	18.6–28.7	18.7	17.1–22.1	28.4	25.3–32.5	<0.0001
Fat mass, kg	3.2–61.2	19.7	15.5–23.2	21.4	17.0–26.7	17.8	13.6–22.2	0.0003
Extra-cellular water, kg	8.7–46.3	15.5	13.2–17.4	12.9	11.7–14.9	16.6	15.3–19.1	<0.0001
24-h UCr/Body weight, mg/kg	8.0–26.3	15.8	12.8–18.0	13.9	11.4–15.8	17.3	15.1–19.8	<0.0001
24-h UCr/ Body cell mass, mg/kg	23.7–70.7	45.8	39.2–52.3	44.7	39.1–51.9	46.4	41.5–52.4	0.40

**Table 2:** Multiple linear regression modeling (stepwise) for creatinine excretion (mg/24 h) based on body cell mass (BCM), age, body weight, serum creatinine, gender, height. VIF (Variance inflation factor). Coefficient of determination *r*^2^ = 0.7454, multiple correlation coefficient = 0.8634.

Independent Variables	Coefficient	Standard Error	Rpartial	*t*	Probability	VIF
Constant	522					
BCM, kg	14.99	4.50	0.256	3.332	0.0011	4.582
Age, years	−7.56	1.16	−0.459	−6.513	<0.0001	1.472
Serum creatinine, mg/dL	−31.76	6.73	−0.351	−4.719	<0.0001	1.086
Body weight, kg	8.54	1.73	0.365	4.934	<0.0001	2.575
Gender, male = 1, female = 0	169.91	39.41	0.324	4.311	<0.0001	2.019

Variables not included in the model: height, body mass index.

**Table 3:** Predicted and measured values of urinary creatinine excretion (UCr), creatinine clearance (CCr) and the glomerular filtration rate (GFR), and mean prediction errors (MPE). MR-BCM CCr (prediction from multiple regression analysis and body cell mass), C&G CCr (Cockcroft–Gault formula), MDRD GFR (prediction of GFR from the Modification of Diet in Renal Disease formula) Median values and interquartile ranges (IQR 25–75) are reported separately for women and men. The statistical significance of the difference between women and men is reported.

Parameters	All Patients *n* = 165			Women *n* = 72		Men *n* = 93		Statistical Significance
	Range	Median	IQR 25–75	Median	IQR 25–75	Median	IQR 25–75	*p*
Measured UCr, mg	444–2102	1077	842–1337	858	713–1020	1261	1085–1504	<0.0001
MR-BCM-UCr, mg	520–1883	1073	869–1328	853	754–985	1305	1139–1450	<0.0001
MPE, mg	175			163		184		
Measured CCr, mL/min/1.73 m^2^	5.4–109.6	53.1	30.9–76.8	53.3	38.5–73.8	52.7	24.0–83.6	0.839
MR-BCM CCr, mL/min/1.73 m^2^	5.1–107.0	54.4	30.3–75.4	55.0	36.6–72.6	55.0	36.6–72.6	0.903
MPE, mL/min/1.73 m^2^	8.88			11.2		6.6		
C&G CCr, mL/min/1.73 m^2^	7.0–118.5	53.4	30.4–78.0	53.7	38.1–73.5	50.9	25.0–83.0	0.427
MPE, mL/min/1.73 m^2^	9.72			12.2		7.3		
GFR, mL/min/1.73 m^2^	3.5–134.4	51.2	25.2–73.7	52.7	33.1–69.4	39.4	20.1–74.9	0.422
BCM GFR, mL/min/1.73 m^2^	0.2–107.6	51.4	29.2–71.5	52.2	34.2–66.5	46.9	22.5–74.3	0.9790
MPE, mL/min/1.73 m^2^	12.4			13.4		11.6		
MDRD GFR, mL/min/1.73 m^2^	4.1–111.0	47.7	27.2–66.5	47.9	31.4–61.2	46.9	19.6–70.1	0.627
MPE, mL/min/1.73 m^2^	12.5			14.4		10.9		

**Table 4:** Accuracy of the glomerular filtration rate (GFR) predicted from body cell mass (BCM GFR) and with the MDRD formula (MDRD GFR) as indicators of different degrees of GFR impairment. Significance of the differences between the area under the curve (AUC) of the Receiver Operating Characteristics (ROC) plots.

	BCM GFR, AUC	Cut-Off	Sensitivity Specificity	MDRD GFR, AUC	Cut-Off	Sensitivity Specificity	*p*
GFR <90 mL/min/1.73 m^2^	0.858	55.5	59.5 100	0.832	55.6	62.8 91.7	0.175
GFR <60 mL/min/1.73 m^2^	0.947	55.5	87.8 92.5	0.961	52.4	87.8 92.5	0.184
GFR <45 mL/min/1.73 m^2^	0.981	51.1	94.9 91.9	0.984	45.9	94.9 93.0	0.630
GFR <30 mL/min/1.73 m^2^	0.987	37.5	97.9 92.3	0.982	33.1	94.8 93.2	0.232
GFR <15 mL/min/1.73 m^2^	0.998	21.4	100 97.1	0.995	15.5	96.3 98.6	0.120

## Appendix A

Examples of calculations of the estimates of urinary creatinine and creatinine clearance from multiple regression correlation, and of the prediction of the glomerular filtration rate from body cell mass and serum creatinine.

Patient # 12 woman, years 51, BW 58.3 kg, BCM = 18.9 kg; SCr = 1.1 mg/dL; measured UCr = 1043 mg, measured CCr = 66.0 mL/min, measured GFR = 52.9 mL/min;

Prediction of UCr: 522 + 14.99 × 18.9 – 7.56 × 51 – 31.76 × 1.1 + 8.54 × 58.3 = 882 mg;

Prediction of CCr: 882/(0.011 × 1440) = 55.7 mL/min;

Prediction of BCM GFR: −5.1 + 3.3 × 18.9/1.1 = 51.6 mL/min.

Patient #16 man, 74 years, BW 91.5 kg, BCM = 31.5 kg, SCr = 2.63 mg/dL, measured UCr = 1844 mg, mCCr = 48.7 mL/min, mGFR = 31.2 mL/min;

Prediction of UCr: 522 + 14.99 × 31.5 − 7.56 × 74 − 31.76 × 2.63 + 8.54 × 91.5 + 16939 = 1303 mg;

Prediction of CCr: 1303/(0.0263 × 1440) = 34.4 mL/min;

Prediction of GFR: −5.1 + 3.3 × 31.5/2.63 = 38.9 mL/min;

The values of clearances and GFR are then reported to the standard BSA of 1.73 m^2^.

## References

[1] Prigent A. (2007). Monitoring renal function and limitations of renal function tests. Semin. Nucl. Med..

[2] Gabriel R. (1986). Time to scrap creatinine clearance?. Br. Med. J..

[3] Greenblatt D.J., Ransil B.J., Harmatz J.S., Smith T.W., Duhme D.W., Koch-Weser J. (1976). Variability of 24-hour urinary creatinine excretion by normal subjects. J. Clin. Pharmacol..

[4] Cockcroft D.W., Gault M.H. (1976). Prediction of creatinine clearance from serum creatinine. Nephron.

[5] Levey A.S., Coresh J., Greene T., Stevens L.A., Zhang Y.L., Hendriksen S., Kusek J.W., Van Lente F. (2006). Chronic Kidney Disease Epidemiology Collaboration. Using standardized serum creatinine values in the modification of diet in renal disease study equation for estimating glomerular filtration rate. Ann. Intern. Med..

[6] Chertow G.M., Lowrie E.G., Wilmore D.W., Gonzales J., Lew N.L., Ling J., Leboff M.S., Gottlieb M.N., Huang W., Zebrowski B. (1995). Nutritional assessment with bioelectrical impedance analysis in maintenance hemodialysis patients. J. Am. Soc. Nephrol..

[7] Lukaski H.C., Bolonchuk W.W., Hall C.B., Siders W.A. (1986). Validation of tetrapolar bioelectrical impedance method to assess human body composition. J. Appl. Physiol..

[8] Cooper B.A., Aslani A., Ryan M., Zhu F.J.-P., Ibels L.S., Allen B.J., Pollock C.A. (2000). Comparing different methods of assessing body composition in end-stage renal failure. Kidney Int..

[9] Donadio C., Consani C., Ardini M., Bernabini G., Caprio F., Grassi G., Lucchesi A., Nerucci B. (2005). Estimate of body water compartments and of body composition in maintenance hemodialysis patients: Comparison of single and multifrequency bioimpedance analysis. J. Ren. Nutr..

[10] Forbes G.B., Bruining G.J. (1976). Urinary creatinine excretion and lean body mass. Am. J. Clin. Nutr..

[11] Lukaski H.C. (1987). Methods for the assessment of human body composition: Traditional and new. Am. J. Clin. Nutr..

[12] Donadio C., Lucchesi A., Tramonti G., Bianchi C. (1997). Creatinine clearance predicted from body cell mass is a good indicator of renal function. Kidney Int..

[13] Donadio C., Lucchesi A., Tramonti G., Bianchi C. (1998). Prediction of creatinine clearance from body composition analysis and plasma creatinine. Ren. Fail..

[14] Donadio C., Consani C., Ardini M., Caprio F., Grassi G., Lucchesi A. (2004). Prediction of glomerular filtration rate from body cell mass and plasma creatinine. Curr. Drug Discov. Technol..

[15] Bianchi C., Donadio C., Tramonti G. (1981). Noninvasive methods for the measurement of renal function. Nephron.

[16] Bianchi C., Bonadio M., Donadio C., Tramonti G., Figus S. (1979). Measurement of glomerular filtration rate in man using DTPA-Tc99m. Nephron.

[17] Lin L.I. (1989). A concordance correlation coefficient to evaluate reproducibility. Biometrics.

[18] Bland J.M., Altman D.G. (1986). Statistical methods for assessing agreement between two methods of clinical measurement. Lancet.

[19] Meng X.L., Rosenthal R., Rubin D.B. (1992). Comparing correlated correlation coefficients. Psychol. Bull..

[20] Altman D.G. (1991). Practical Statistics for Medical Research.

[21] Lalonde R.L., Pao D. (1984). Correlation coefficient versus prediction error in assessing the accuracy of digoxin dosing methods. Clin. Pharm..

[22] Tape T.G. Interpreting Diagnostic Tests. http://gim.unmc.edu/dxtests/roc3.htm.

[23] Patel S.S., Molnar M.Z., Tayek J.A., Ix J.H., Noori N., Benner D., Heymsfield S., Kopple J.D., Kovesdy C.P., Kalantar-Zadeh K. (2013). Serum creatinine as a marker of muscle mass in chronic kidney disease: Results of a cross-sectional study and review of literature. J. Cachexia Sarcopenia Muscle.

[24] Hull J.H., Hak L.J., Koch G.G., Wargin W.A., Chi S.L., Mattocks A.M. (1981). Influence of range of renal function and liver disease on predictability of creatinine clearance. Clin. Pharmacol. Ther..

[25] Rolin H.A., Hall P.M., Wei R. (1984). Inaccuracy of estimated creatinine clearance for prediction of iothalamate glomerular filtration rate. Am. J. Kidney Dis..

[26] Hossain M.A., Attia A., Shoker A. (2010). Measurement error in estimated GFR slopes across transplant chronic kidney disease stages. Am. J. Nephrol..

[27] Botev R., Mallié J.P., Wetzels J.F., Couchoud C., Schück O. (2011). The clinician and estimation of glomerular filtration rate by creatinine-based formulas: Current limitations and quo vadis. Clin. J. Am. Soc. Nephrol..

[28] El-Minshawy O., El-Bassuoni E. (2013). Validity of current equations to estimate glomerular filtration rate in kidney transplant recipients. Transplant. Proc..

[29] Perico N., Gaspari F., Remuzzi G. (2005). Assessing Renal Function by GFR Prediction Equations in Kidney Transplantation. Am. J. Transplant..

[30] Mitch W.E., Collier V.U., Walser M. (1980). Creatinine metabolism in chronic renal failure. Clin. Sci..

[31] Donadio C., Kanaki A., Donadio E., Tognotti D. (2010). Assessment of nutritional status and risk of death in maintenance haemodialysis patients. Healthmed.

[32] Flury S., Trachsler J., Schwarz A., Ambühl P.M. (2015). Quantification of excretory renal function and urinary protein excretion by determination of body cell mass using bioimpedance analysis. BMC Nephrol..

[33] Donadio C., Ardini M., Bernabini G., Caprio F., Consani C., Grassi G., Timio M., Wizemann V., Venanzi S. (2005). Prediction of glomerular filtration rate in overweight and obese chronic kidney disease patients. 12th Meeting in Cardionephrology.

